# Various Aspects of Calcium Signaling in the Regulation of Apoptosis, Autophagy, Cell Proliferation, and Cancer

**DOI:** 10.3390/ijms21218323

**Published:** 2020-11-06

**Authors:** Simone Patergnani, Alberto Danese, Esmaa Bouhamida, Gianluca Aguiari, Maurizio Previati, Paolo Pinton, Carlotta Giorgi

**Affiliations:** 1 Department of Medical Sciences, Laboratory for Technologies of Advanced Therapies, University of Ferrara, 44121 Ferrara, Italy; simone.patergnani@unife.it (S.P.); alberto.danese@unife.it (A.D.); esmaa.bouhamida@unife.it (E.B.); 2 Department of Biomedical and Surgical Specialty Sciences, University of Ferrara, 44121 Ferrara, Italy; gianluca.aguiari@unife.it; 3 Department of Morphology, Surgery and Experimental Medicine, Section of Human Anatomy and Histology, Laboratory for Technologies of Advanced Therapies (LTTA), University of Ferrara, 44121 Ferrara, Italy; maurizio.previati@unife.it

**Keywords:** calcium, cancer, apoptosis, autophagy, cell cycle, therapy, chemotherapy

## Abstract

Calcium (Ca^2+^) is a major second messenger in cells and is essential for the fate and survival of all higher organisms. Different Ca^2+^ channels, pumps, or exchangers regulate variations in the duration and levels of intracellular Ca^2+^, which may be transient or sustained. These changes are then decoded by an elaborate toolkit of Ca^2+^-sensors, which translate Ca^2+^ signal to intracellular operational cell machinery, thereby regulating numerous Ca^2+^-dependent physiological processes. Alterations to Ca^2+^ homoeostasis and signaling are often deleterious and are associated with certain pathological states, including cancer. Altered Ca^2+^ transmission has been implicated in a variety of processes fundamental for the uncontrolled proliferation and invasiveness of tumor cells and other processes important for cancer progression, such as the development of resistance to cancer therapies. Here, we review what is known about Ca^2+^ signaling and how this fundamental second messenger regulates life and death decisions in the context of cancer, with particular attention directed to cell proliferation, apoptosis, and autophagy. We also explore the intersections of Ca^2+^ and the therapeutic targeting of cancer cells, summarizing the therapeutic opportunities for Ca^2+^ signal modulators to improve the effectiveness of current anticancer therapies.

## 1. Introduction: A General Overview of Ca^2+^ Signaling

**Figure 1:**
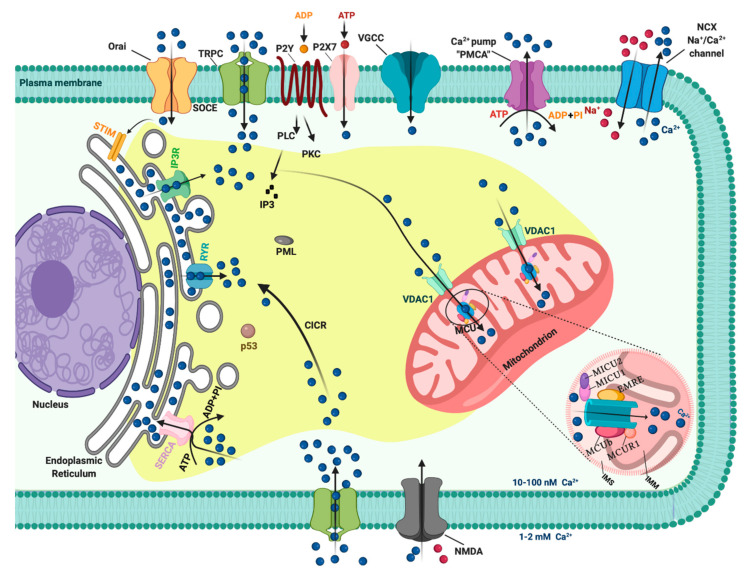
The intracellular Calcium (Ca^2+^) signaling. Different Ca^2+^ transporters, channels, exchangers, binding/buffering proteins and pumps mediate the regulation of cytosolic Ca^2+^ concentration. In the plasma membrane (PM), PM Ca^2+^-ATPases (PMCA) pumps, transient receptor potential channels (TRPC), voltage-gated Ca^2+^ channels (VGCC), Na^+^/Ca^2+^ exchanger (NCX), and purinergic P2 receptors regulate the transport of Ca^2+^ ions inside and outside cells. Inositol 1,4,5-triphosphate receptors (IP3R), ryanodine receptors (RyR), and sarcoendoplasmic reticulum Ca^2+^-ATPase (SERCA) pumps control the storage of Ca^2+^ in the endoplasmic reticulum. Finally, voltage-dependent anion channels (VDAC) and members of the mitochondrial Ca^2+^ uniporter family are critical for controlling the mitochondrial Ca^2+^ uptake. Created with BioRender.com.

In resting cells, the intracellular free Ca^2+^ concentration ([Ca^2+^]_i_) is maintained at lower levels than extracellular fluid. Indeed, there is a 20,000-fold gradient between outside (about 1.2 mM) and inside (approximately 10–100 nM) of cells. Moreover, in the mitochondria and in the nucleus, the concentrations of Ca^2+^ are similar to those in the cytoplasm. In the endoplasmic reticulum (ER), considered the main intracellular Ca^2+^ store, the [Ca^2+^] ranges between 100 and 800 μM [1]. In addition, direct measurements of Ca^2+^ levels show that lysosomes present an internal [Ca^2+^] of about ≈500 μM [2]. Therefore, it exists an elaborate system of Ca^2+^-transporters, -channels, -exchangers, -binding/buffering proteins, and -pumps that finely regulate Ca^2+^ flow inside and outside of cells and among intracellular organelles [3]. This network permits preservation of a low resting [Ca^2+^] and regulates the propagation of intracellular Ca^2+^ changes that are fundamental to intracellularly transmitted biological information and important physiologic processes, including metabolism, cell proliferation and death, protein phosphorylation, gene transcription, neurotransmission, contraction, and secretion [4,5]. During cell stimulation the [Ca^2+^]_i_ can increase more than twofold at the micromolar level. Different channels situated in the plasma membrane (PM) induce the influx of extracellular Ca^2+^ into the cells. Among these channels, the most important are transient receptor potential channels (TRPC) [6], store-operated Ca^2+^ entry (SOCE) channels such as ORAI and STIM [7], voltage-gated Ca^2+^ channels (VGCC) in excitable cells [8], receptor-operated Ca^2+^ channels such as the N-methyl-d-aspartate receptor (NMDA) [9] and purinergic P2 receptors [10], whose activation determines cytosolic Ca^2+^ influx. Intracellular Ca^2+^ increases may be also due to Ca^2+^ release from internal stores, mainly via inositol 1,4,5-triphosphate receptors (IP3Rs) situated on the ER [11,12]. IP3Rs are large-conductance cation channels that are activated in response to the activation of cell surface receptors [13]. Despite different physiological and pharmacological profiles, ryanodine receptors (RyRs) have an approximatively 40% homology with IP3Rs and are the Ca^2+^ release channels on the sarcoplasmic reticulum of muscle cells [14]. A prolonged elevation of [Ca^2+^]_i_ has adverse effects for the cells. Therefore, different channels, pumps, and buffering systems reestablish low [Ca^2+^]_i_. The reuptake of Ca^2+^ into the ER lumen is allowed by the activity of sarcoendoplasmic reticulum Ca^2+^-ATPase (SERCA), which pumps Ca^2+^ into the ER with a stoichiometry of 2:1 Ca^2+^/ATP and by the secretory protein calcium ATPase (SPCA), which transports Ca^2+^into the Golgi apparatus [15]. Plasma membrane Ca^2+^ transport ATPase (PMCA) and Na^+^/Ca^2+^ exchanger (NCX) are the two mechanisms situated on the PM responsible for Ca^2+^ extrusion. PMCA is a pump that belongs to the class of P-type ATPases that pump Ca^2+^ across the PM out of the cell at the expense of ATP [16,17]. NCX permits Ca^2+^ extrusion against its gradient without energy consumption by using the electrochemical gradient of Na^+^. For each Ca^2+^ ion extruded, three Na^+^ ions enter the cell [18]. Additionally, mitochondria significantly contribute to the signaling pattern of released intracellular Ca^2+^. Indeed, these organelles may act as Ca^2+^ buffers [19]. It is widely accepted that Ca^2+^ entry into mitochondria is mediated by the activity of the mitochondrial calcium uniporter (MCU) complex, composed of the pore-forming subunit of the MCU channel together with several regulatory proteins (MICU1, MICU2, MICU3, MCUR1, MCUb, and EMRE) [20]. Advances in the studies regarding Ca^2+^ dynamics have revealed that a network of membrane contact sites has a determinant role in Ca^2+^ signaling. These contacts create microdomains that permit the exchange of metabolites and signals between membranes of different compartments. The structural and functional interactions between the ER and mitochondria (the mitochondria associated membranes, MAMs) represent the main central hub for controlling Ca^2+^ exchange between these two compartments [21]. Disruption of MAMs result in the suppression of ER Ca^2+^-release and alters mitochondrial Ca^2+^ accumulation (Figure 1). ER membranes are also interconnected with the membranes of lysosomes to form the ER-lysosome membrane contact sites. It has been proposed that the IP3R-mediated ER release of Ca^2+^ is a mechanism for mediating the reestablishment of Ca^2+^ levels in lysosomes [22]. However, the Ca^2+^ transporter mediating this Ca^2+^ transmission remains unidentified. In contrast, the identity of channels regulating lysosomal Ca^2+^ release has been established. Several channels mediate this Ca^2+^ transport. Among these channels, the mucolipin subgroup of the TRP ion channel family, in particular the isoform TRPML1, represents the most well-established lysosomal Ca^2+^ release channels [23].

Overall, all these mechanisms preserve the correct Ca^2+^ homeostasis of the cell and regulate the spatiotemporal patterning of the Ca^2+^ signal. Any alterations to this highly connected network of Ca^2+^ transporters, channels, exchangers, binding/buffering proteins, and pumps determine the unregulated Ca^2+^ dynamics that affect almost every aspect of cell function, such as proliferation, gene expression, cell death, and protein phosphorylation and dephosphorylation [24]. There is evidence that cancer cells have disrupted Ca^2+^ signaling, where the expression of Ca^2+^ channels/pumps and Ca^2+^-regulating proteins is altered [3]. Therefore, remodeling of these derailed Ca^2+^ features may be a potential target for cancer therapies. In view of this possibility, we outline the contributions of Ca^2+^ signaling to the cell cycle and cell proliferation, apoptosis, and autophagy with particular attention to the cancer context. We also focus on the potential impact of Ca^2+^ signal modulation in cancer therapy.

## 2. Ca^2+^ Homeostasis during Cell Cycle and Tumor Growth

In recent years, the importance of cell cycle progression regulating by Ca^2+^ signals has been recognized, especially upon the development of probes that allow a very sensible and direct visualization of Ca^2+^ transients. Spontaneous Ca^2+^ oscillations at the three major cell cycle checkpoints have been described. For example, transient [Ca^2+^]_i_ increases during the G1/S phase transition [25], the G2/M transition [26], and the metaphase to anaphase transition [27]. Ca^2+^ is also required in the early G1 phase, when cells re-enter the cell cycle, to promote the activation of c-AMP-responsive element binding protein, AP1 (FOS and JUN) transcription factors, and the nuclear factor of activated T-cell (NFAT) [28]. The cell cycle is principally controlled by the expression of protein complexes organized around cyclin-dependent protein kinases (CDKs), which coordinate the entry into the next phase of the cell cycle only when bound to a cyclin. The essential bridge between Ca^2+^ ions and CDK/cycline complexes is undoubtedly represented by the Ca^2+^-sensors calmodulin (CAM) and calcineurin (CaN). These Ca^2+^-binding proteins, and intermediary proteins such as Ca^2+^/calmodulin-dependent protein kinases (CAMKI, CAMKII, and CAMKIII), interact with CDK/cycline complexes regulating crucial cell cycle events, including DNA synthesis (i.e., by cyclin D1-CDK4 regulation through CAMKI) [29], microtubule stability regulation, e.g., by decreasing the amount of Ca^2+^ required for microtubule depolymerization [30] and by interacting with nucleoporin p62 [31] and for cytokinesis completion [32]. The essential role played by Ca^2+^ as a regulator of cell cycle progression has been extensively presented in publications based on the use of CAM activity inhibitors. In particular, treatment with W-7 and W-13 CAM antagonists induced G1 phase cell cycle arrest by downregulating cyclins and upregulating p21 [33]. Moreover, the microinjection of monoclonal antibodies against CAM inhibited the synthesis of DNA in a dose-dependent manner [34]. One of the most fascinating aspects of studying these cell cycle progression regulation mechanisms is undoubtedly the detection of new potential targets in the fight against cancer. Indeed, the most important characteristic of cancer cells is certainly their ability to undergo biological changes that sustain their unlimited replicative capacities. The behavioral study of Ca^2+^ channels and pumps in relation to the cell cycle and proliferation has turned out to be very important, especially in recent years. Some examples of how a perturbation of Ca^2+^ signaling can lead to cell cycle dysregulation with consequent repercussions on tumor pathologies are worthy of description. Cytosolic Ca^2+^ levels modulate guanosine exchange factor and GTPase activating protein, which are a RAS stimulator and a RAS inhibitor, respectively. RAS, in turn, stimulates the proliferative mitogen-activated protein kinase (MAPK) pathway, which initiates the cells transition into the S phase because of phosphorylation of the tumor suppressor RB1 upon cytoplasmic cyclin D1 upregulation. Constitutively high cytosolic Ca^2+^ levels in cancer cells can lead to uncontrolled growth through the removal of the G1/S transition checkpoint [35]. ORAI3, a SOCE component, is overexpressed in breast cancer biopsy samples and is involved in breast cancer cell proliferation and cell cycle progression by modulating the G1 phase and G1/S transition regulator protein activity [36]. ORAI3 is an upstream regulator of c-myc that controls the cell cycle and proliferation in breast cancer by modulating the expression of cyclins D1 and E, CDKs 4 and 2, cyclin-dependent kinase inhibitor p21, and tumor-suppressing protein p53 [37]. A large number of studies have indicated key roles for cyclin D and cyclin E expression in breast cancer cell cycle deregulation; in fact, cyclins D1 and E proteins are overexpressed in more than 50% of breast tumors [38,39,40]. Additionally, VGCCs are associated with cell proliferation regulation. Specific VGCCs family genes were downregulated in breast, kidney, brain, and lung cancers, showing that these Ca^2+^ channels play roles as tumor suppressor genes [41]. On the other hand, members of the VGCCs family are expressed at detectable levels in melanoma cells but not in untransformed melanocytes, and the use of T-type channel inhibitors induces cell cycle arrest with a significant increase of the percentage of cells in the G1 phase and a reduction of cells in the S phase [42].

Changes in the expression of TRPCs have been implicated in prostate cancer. In particular, transient receptor potential vanilloid subfamily member 6 (TRPV6) in prostate cancer reduces the activation of NFAT and decreases cell accumulation in the S phase of the cell cycle [43].

It has been reported that CAMKs expression alterations have repercussions on cell cycle progression in several tumor pathologies. Parmer et al. described CAMKIII as a potential pharmacological target against glioma because of its important link to cell proliferation, viability, and malignancy [44]. Chemical inhibition of CAMKIII resulted in the reduction of growth of glioma cells line, which was mirrored by a blocked G1 phase transition in the cell cycle. In breast cancer, CAMKIII activation leads to the phosphorylation of elongation factor-2 and transient inhibition of protein synthesis. These events are controlled by mitogens and are predominant in the S phase of the cell cycle [45]. CAMKII has been described to be crucial in T cell lymphoma cell proliferation; its genetic ablation drives a significant increase in the percentage of G2/M phase cells and a decrease in the percentage of S phase cells, outcomes that are consistent with the inhibition of cell proliferation [46].

## 3. Role of Ca^2+^ in Apoptosis and Cancer

**Figure 2:**
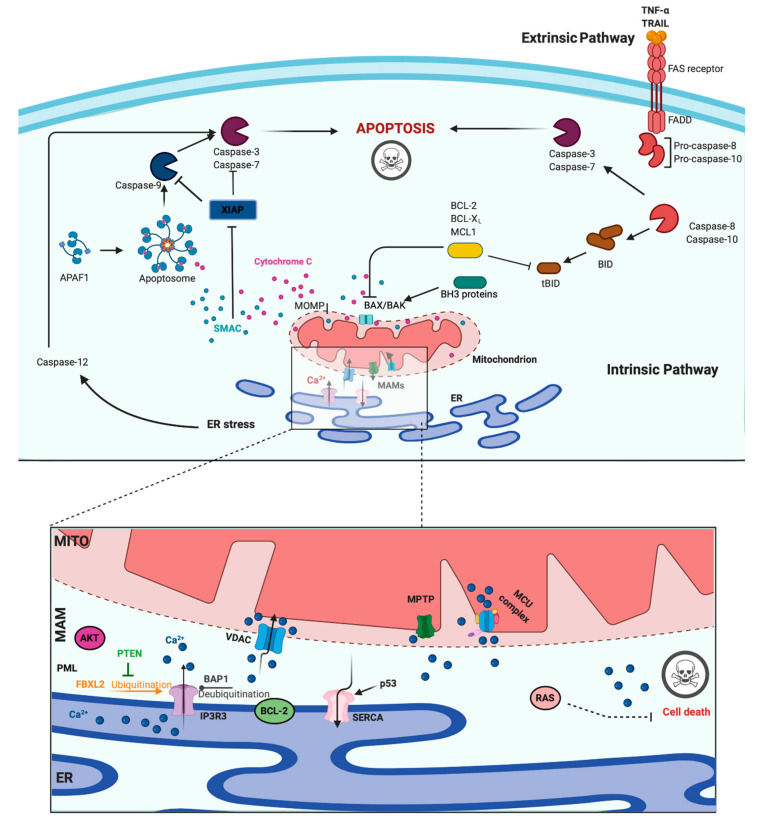
Apoptosis and Calcium (Ca^2+^) dynamics in cancer. Apoptosis is the best-characterized and studied programmed cell death. In the extrinsic pathway, extracellular ligands determine the formation of the death-inducing signaling complex that activates the caspases cascade. The intrinsic apoptotic pathway is characterized by permeabilization of the mitochondria that allows the release of cytochrome c (cyt-c) and other apoptogenic factors in the cytosol. Once released, these factors bind apoptotic protease activating factor 1 (APAF1) and form a multiprotein complex called the apoptsome that recruits and activates the caspases. Ca^2+^ has a major role during intrinsic apoptosis, and excessive mitochondrial Ca^2+^ accumulation may trigger apoptosis. Different proteins were found to control apoptotic machinery by regulating Ca^2+^ flux between endoplasmic reticulum (ER) and mitochondria. Antiapoptotic B-cell lymphoma-2 (BCL-2) members block apoptotic program by lowering Ca^2+^ levels in the ER, thereby attenuating subsequent Ca^2+^ release. p53 localizes at the ER–mitochondria interface to improve Ca^2+^ dynamics and apoptosis by increasing sarcoendoplasmic reticulum Ca^2+^-ATPase (SERCA) pumps activities. Additionally, the tumor suppressors promyelocytic leukemia protein (PML), BRCA1-associated protein 1 (BAP1), and phosphatase and tensin homolog (PTEN) move to the mitochondria associated membranes (MAMs) to regulate Ca^2+^-dependent apoptosis. They determine the activation of Ca^2+^ release from the ER by modulating the activity of inositol 1,4,5-triphosphate receptor 3 (IP3R3). Mutations or loss of these tumor suppressors are frequently found in diverse human tumor samples, where they lead to a reduction in Ca^2+^ homeostasis and the apoptosis rate, favoring cellular proliferation, tumor growth, maintenance, and metastasis. Created with BioRender.com.

A main hallmark of cancer cells is evasion of programmed cell death (PCD) [47]. PCD is a genetically determined cell routine in which cells undergo an unexpected decline in homeostasis and functionality, triggering several intracellular pathways and ultimately cell death. Different types of PCD exists. During viral or microbial infections, PCD is a part of the host immune response, with traits similar to those of apoptosis and necrosis in a process referred to as pyroptosis or necroptosis. Pyroptosis is a caspase-dependent form of PCD that leads to membrane permeabilization and cell swelling through gasdermin D activation. Pyroptosis is triggered via inflammasome that was induced when the cell senses changes after viral or microbial invasion and is linked to atherosclerosis, metabolic disease, and neuroinflammatory disorders [48,49]. Another type of PCD is necroptosis, an inflammation-dependent form of PCD, which is similar to necrosis in the absence of caspase involvement and eventual membrane permeabilization, cell swelling, and lysis, with the subsequent leakage of a plethora of proinflammatory molecules. [50]. However, the best-characterized form of PCD is apoptosis. Apoptosis is a strictly controlled phenomenon, typically manifested by chromatin condensation, DNA and nuclear fragmentation, mitochondrial failure, proteolytic enzyme activation and membrane blebbing, even with cell membrane integrity. The cell membrane forms the interface with cell-impermeant stimuli, which interact with membrane receptors triggering the so-called intrinsic pathways. Thus, membrane-induced apoptosis depends upon extracellular ligands, such as tumor necrosis factor-α (TNFα) and first apoptosis signal (FAS) ligand, and their receptors, TNFRs and FAS. Employing several adaptors, some of which carry the death effector domain, these activated receptors induce the formation of the death-inducing signaling complex (DISC). DISC stimulates the autoproteolytic cleavage of initiator caspase-8, which in turn activates the executioner caspase-3, -6, and -7. This proteolytic cascade strongly amplifies the initial signal and initiates the cleavage of hundreds of cellular targets and is thus crucial for the main morphological features of apoptosis [51] (Figure 2, upper panel).

In addition to the extracellular-driven intrinsic pathway, an intracellular, mitochondria-centered pathway is activated in apoptosis, with the function of coupling insurmountable mitochondrial stress to cell death. Nevertheless, a wide number of stress conditions, including hypoxia, alteration, or poisoning of the electron transfer chain, unbuffered ROS production, and imbalanced mitochondrial protein homeostasis, can initiate mitochondrial permeability transition (MPT) [52,53]. MPT, followed by mitochondrial osmotic imbalance and mitochondrial outer membrane permeabilization, allows the release of several mitochondrial proteins, such as cytochrome C (cyt-C). In particular, cytoplasmic cyt-C binds to apoptotic protease activating factor 1 to form a multiprotein complex able to recruit and activate the initiator caspase-9 via the caspase recruitment domain. Caspase-9 in turn cleaves and activates the other executioners, namely, caspase-3, -6, and -7 [51]. An important regulatory mechanism of the intrinsic pathway is the mitochondrial Ca^2+^ load. Ca^2+^ is an important regulator of Krebs cycle dehydrogenases [54] and normally accumulated in the mitochondrial matrix at concentrations 10-fold higher than those measured in the cytosol. Under specific conditions, Ca^2+^ overload can trigger MTP by opening the permeability transition pore with the consequent release of apoptogenic factors [52,55] (Figure 2, upper panel). During carcinogenesis, cancer cells use different machinery to circumvent apoptosis and acquire a profound survival and proliferative advantage. They accumulate genetic alterations that increase or decrease the expression of pro- and/or antiapoptotic genes. Moreover, cancer cells can prevent apoptosis through post-translation modification, such as phosphorylation/dephosphorylation. In contrast, cancer cells may also evade apoptosis by reducing the Ca^2+^ signaling necessary to prompt the apoptotic machinery. The latest evidence shows that Ca^2+^ release from the ER is the main mechanism regulating the mitochondrial Ca^2+^ remodeling and apoptosis [56]. The first observation was obtained by studying B-cell lymphoma-2 (BCL-2) proteins. These proteins are classified into antiapoptotic category (BCL-2, BCL-xL, and Mcl-1) and a pro-apoptotic category (like Bax, Bak, Bim, Bid, etc.). Evidence demonstrates that antiapoptotic BCL-2 proteins regulate the apoptotic program by controlling ER-mitochondrial Ca^2+^ transfer in both organelles, and in particular, recent studies indicate that these proteins also exert antiapoptotic functions at MAMs levels [57]. Overexpression of pro-apoptotic BCL-2 proteins was found to reduce both ER-Ca^2+^ release either by direct control of IP3R3-induced pore opening or by lowering the Ca^2+^ content of the ER [58,59]. As a consequence, Ca^2+^-induced MPT is prevented, and the apoptotic program is abolished. Furthermore, it has also been demonstrated that BCL-2, BCL-XL, and Mcl-1 determine pro-survival IP3Rs-mediated Ca^2+^ oscillations that are necessary to increase mitochondrial energy production and stimulate cell proliferation [60]. Overall, these proteins impact three important aspects of cancer development: cell death, survival, and energy production. Consistently, upregulation of pro-apoptotic BCL-2 members was found in different human cancer samples and was associated with the invasion and metastasis of colon, breast, and gastric cancer [61]. BCL-2 members are not the only proteins that regulate apoptosis and cell proliferation by modulating the ER-Ca^2+^ release into mitochondria. The oncogene RAS plays a pivotal role in tumor growth and maintenance of the tumor environment [62]. To exert this function, RAS deregulates ER Ca^2+^ dynamics with the consequent inhibition of apoptosis, impairment to mitochondrial metabolism, and promotion of malignant cell survival [63]. Additionally, the oncogene AKT phosphorylates and inactivates several proteins (such as Bad, Bax, and hexokinase-2) that normally work to promote the Ca^2+^-dependent apoptotic response. Furthermore, AKT inhibits the apoptotic process by exerting a direct control of IP3R3 opening, thus avoiding the Ca^2+^ overload necessary to activate the intrinsic apoptosis [64]. If oncogene proteins promote cell survival and proliferation by blocking Ca^2+^-mediated apoptosis, it is not surprising that tumor suppressors activate the same mechanism. Protein phosphatase and tensin homolog (PTEN), which is frequently lost or mutated in several cancers, counteracts the activity of AKT and restores Ca^2+^ transfer and reestablishes subsequent cell death [65]. In addition, PTEN was also recently found to block the proteosomal degradation of IP3R3 provoked by the F-box protein FBXL2 [66]. The activity of AKT is also balanced by the tumor suppressor promyelocytic leukemia protein (PML), which, together with IP3R3, AKT, and the phosphatase PP2a, creates a complex that rules ER-mitochondria Ca^2+^ transfer [67]. BRCA1-associated protein 1 (BAP1) is a tumor suppressor frequently mutated in diverse malignancies, especially in mesotheliomas, for which alteration of Ca^2+^ dynamics had previously described [68]. BAP1 works as a deubiquitinating enzyme and is involved in different processes, such as DNA repair and transcription. Recently, it has been demonstrated that BAP1 also deubiquitylates and stabilizes IP3R3. Therefore, following DNA damage exposure, cells can undergo to apoptosis by activating ER-mitochondria Ca^2+^ transfer [69]. Additionally, p53 regulates tumorigenesis by modulating ER-mitochondria Ca^2+^ flux. In this case, the tumor suppressor was found to improve intracellular Ca^2+^ accumulation by increasing SERCA pump activities [70] (Figure 2, lower panel). Apart from the well-established roles for ER-Ca^2+^ dynamics in cancer, recent investigations suggest that impairments in lysosomal Ca^2+^ processes are also important in driving tumorigenesis. Consistent with this finding, cancers of the bladder, head and neck region, and thyroid exhibit increased expression of the gene mucolipin 1 [71], which encodes the lysosomal Ca^2+^ release channel (transient receptor potential mucolipin 1, TRPML1). Consistent with this, TRPML1 inhibition reduces the proliferation of cancer cells [71]. Additionally, the expression of TRPML2 isoforms has been found to be highly expressed in glioma tissues [72]. The transcription factor EB (TFEB) is a master regulator of lysosome function. Altered expression and/or activity of TFEB has been found in pancreatic, kidney, and non-small cell lung cancers [73,74,75] and is associated with aggressive clinical features in colorectal cancer [76]. Interestingly, it has been demonstrated that TFEB activities are highly modulated by a Ca^2+^-enriched microenvironment that is created following lysosomal Ca^2+^ release mediated by TRPML1 channels [77] and that TFEB itself modulates the lysosomal Ca^2+^ buffering capacity [78], thereby suggesting a primary role of lysosomal Ca^2+^ in TFEB-associated cancers. Recent advances in RNA research have revealed that the levels of microRNAs (miRs), a class of small noncoding RNAs that regulate various target genes leading to a decrease in target protein levels, are associated with a variety of human diseases, including cancer. In this context, miRs not only regulate the functions of several oncogenes and tumor suppressors but also target genes that control intracellular Ca^2+^ dynamics. Among these miRNAs, oncogenic miR-25 provokes the downregulation of MCU with subsequent decreases in mitochondrial Ca^2+^ uptake and a reduction in the apoptotic process. Accordingly, prostate and colon cancer cells express increased miR-25 levels and present reduced MCU levels [79]. miR-25-dependent MCU downregulation has also been observed in pulmonary artery smooth muscle cells, where decreases in mitochondrial Ca^2+^ levels cause the activation of a cancer-like phenotype characterized by increased cellular proliferation, migration, and apoptotic resistance. In addition to miR-25, the authors also identified miR-138 as a regulator of MCU expression and demonstrated that nebulizing anti-miR-25 and miR-138 restored MCU expression and abolished the cancer-like phenotype [80]. Another miR involved in cancer is miR-34, whose aberrant expression has been detected in the T lymphocytes of cancer patients [81,82]. It has been observed that miR-34 is also a regulator of SOCE by targeting the expression of IP3R2, STIM1, and ORAI3 in immune cells. These results suggest that miR-34 may control the activities of pro- and antiapoptotic genes by regulating Ca^2+^ signaling, thereby controlling the activation and proliferation of T cells and inducing the inhibition of the antitumor immune response. Finally, several other miRs have been suggested to regulate Ca^2+^ homeostasis and apoptosis. Despite this, a direct correlation between these miRs, Ca^2+^, and cancers has not been demonstrated. Only to cite a few, miR-132 influences Ca^2+^ levels by regulating the expression of the exchanger NCX [83]. MiR-7 reduces voltage-dependent anion channels 1 (VDAC1) expression and diminishes the efflux of Ca^2+^ from mitochondria [84]. MiR-1 regulates the expression of MCU and protects mitochondria from Ca^2+^ overload in cardiac myocytes during development [85].

## 4. The Regulation of Autophagy by Calcium Signals and Its Involvement in Cancer

Autophagy is an intracellular catabolic process that targets and isolates cytoplasmic components, ranging from low-dimension biological macromolecules to whole organelles, and successively enables their delivery to lysosomes for degradation. As a whole, autophagy plays two main roles. First, it is a homeostatic mechanism, ensuring the removal of damaged proteins and organelles. Selective forms of autophagy can specifically target mitochondria (mitophagy), the endoplasmic reticulum (reticulophagy), peroxisomes (pexophagy), lipid droplets (lipophagy), or invading pathogens (xenophagy) [86]. Second, the degraded material is a source of amino acids and lipids for the subsequent de novo synthesis of proteins and lipids. This recycling is of particular importance in the presence of conditions limiting the availability of amino acids, such as during starvation, when the presence of the whole amino acid pool can be guaranteed only through the demolition of cellular proteins, which serve as a reservoir of amino acids. For this reason, autophagy is mainly regarded as a survival mechanism executed during shortage conditions and also during similar stressful circumstances, such as hypoxia or pathogen invasion [87]. Autophagy is initiated with the formation of double-membrane lined vesicles, which gather and fuse, engulfing portions of the cytoplasm. The resulting double-membrane vacuoles are called autophagosomes (APs), which can fuse with vesicles in the endocytic pathway at different stages of maturation or directly with the lysosome, becoming an autolysosome. At this point, the acidic hydrolases break down the macromolecules into smaller constituents that are released back into the cytosol by lysosomal transporters and permeases.

**Figure 3:**
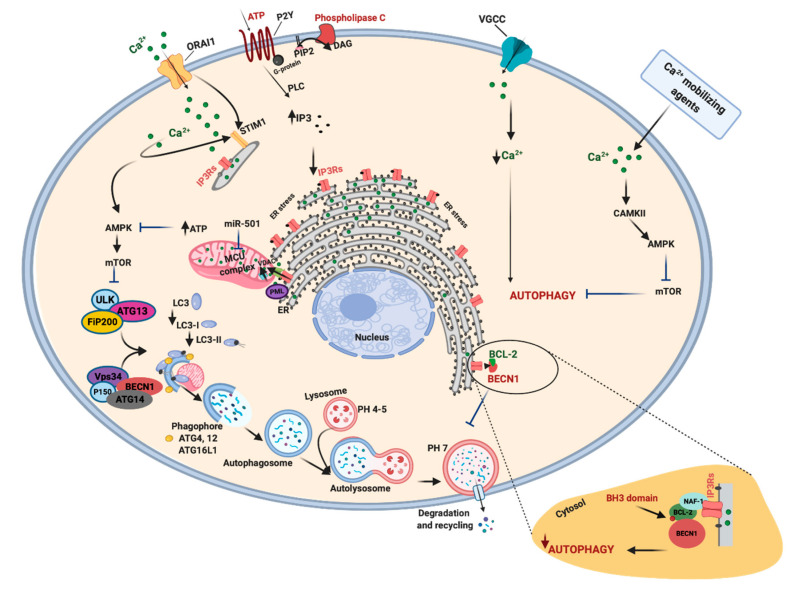
Autophagy, Ca^2+^ and cancer. Autophagy is a key process necessary for the maintenance of the correct cell homeostasis. The unc-51 like autophagy activating kinase 1-2/autophagy-related 13/200-kDa focal adhesion kinase family-interacting protein (ULK/ATG13/FIP200) complex together with other proteins, such as coiled-coil, moesin-like BCL2 interacting protein (BECN1), controls the formation and elongation of autophagosome vesicles. The activity of BECN1 is also regulated by the portion of B-cell lymphoma-2 (BCL-2) pool that is localized in the endoplasmic reticulum (ER). A series of autophagy-related genes (ATG) is essential to the growth and closure of the autophagosome. Additionally, Ca^2+^ signal intervenes to modulate the autophagic machinery. A correct Ca^2+^ transfer between the ER and mitochondria permits optimal mitochondrial Ca^2+^ uptake and consequent ATP production. This signal downregulates the activation of the energy sensor of ATP/AMP ratio, 5′ adenosine monophosphate-activated protein kinase (AMPK), which is the most investigated positive regulator of autophagic induction mechanism, AMPK-ULK1-mammalian target of rapamycin (mTOR). When Ca^2+^ transfer from the ER to mitochondria and/or Ca^2+^ is imported into mitochondria is compromised, AMPK is activated, and survival autophagy is induced. For example, tumors characterized by loss of the tumor suppressor promyelocytic leukemia protein (PML) have enhanced autophagy levels and attenuated Ca^2+^ dynamics. In renal carcinoma, miR-501 decreases the activity of mitochondrial Ca^2+^ uniporter (MCU) channel, provoking a reduction in ATP production and recruitment of AMPK-ULK-mTOR pathway. In contrast, it has been observed that reduced Ca^2+^ dynamics may also activate autophagic cell death. Cancers have increased voltage-gated Ca^2+^ channels (VGCC): their inhibition reduces Ca^2+^ entry and activates autophagy to reduce cell proliferation. A decrease in ORAI1 delays cytoplasmic Ca^2+^ clearance and activates autophagy. Diverse Ca^2+^ mobilizing agents increase in intracellular Ca^2+^ levels and activate pro-survival autophagy by activating Ca^2+^/calmodulin-dependent protein kinases 2 (CAMKII). Created with BioRender.com.

In APs formation, unc-51 like autophagy activating kinase 1-2/autophagy-related 13/200-kDa focal adhesion kinase family-interacting protein (ULK/ATG13/FIP200) complex is the upstream regulator [88]. The ULK1 complex and related adaptor proteins are controlled through the action of kinases such as mammalian target of rapamycin (mTORC) and 5′ adenosine monophosphate-activated protein kinase (AMPK). mTORC is a protein complex that integrates different stimuli involved in nutritional status and oxygen levels to regulate several cellular processes, in particular inhibiting autophagy via direct phosphorylation of ULK1. On the other hand, AMPK stimulates autophagy through ULK1 phosphorylation in response to nutritional deprivation, oxygen unavailability, and mitochondrial dysfunction [89]. Activated ULK1 and 2 proteins, in turn, not only inhibit mTORC and AMPK but also phosphorylate and activate coiled-coil, moesin-like BCL2 interacting protein (BECN1). BECN1 is part of a complex that includes class III phosphatidylinositol 3-kinase (PI3K) and its regulatory proteins, which, when activated, are involved in the nucleation and elongation of the phagophore upon activation. The first step is the synthesis of phosphatidylinositol-3-phosphate (PI3P) by phosphorylation of phosphatidylinositol in the membrane of the ER, mitochondria, Golgi complex, endosomes, or PM [90]. PI3P recruits several adaptor proteins involved in the elongation of a sack-like, omega-shaped structure, which grows and closes around and binding the material to be digested. The BECN1 interactome, formed by BECN1 and various interacting proteins, regulates the nucleation and elongation of phagophore [91]. While PI3P behaves as a positive regulator of autophagy and is involved in the recruitment of various adaptor proteins, BECN1 can be negatively regulated by the antiapoptotic proteins BCL-2, BCL-XL and other members of BCL-2 family. On the one hand, these proteins bind to BECN1 through the BCL-2-homology-3 (BH3) domain and inhibit autophagy by disrupting the interaction between BECN1 and the class III PI3K complex. On the other hand, BCL-2 phosphorylation can attenuate BECN1 sequestration and autophagy inhibition [91]. Two systems, the ATG12-ATG5-ATG16L1 and microtubule-associated proteins 1A/1B light chain 3 (LC3)-phosphatidylethanolamine (PE) complexes, seem to be essential for the growth and closure of the APs. After successive protein interactions, the resulting protein complex joins LC3 at membrane lipid PE. The lapidated complex, bound to the autophagosomal membrane, recruits other adaptor proteins to recognize cargo material, elongate and close the vesicle. The last stage is fusion with lysosomal membrane, followed by lysosomal compartment acidification, decomposition of macromolecules by hydrolases and lipases, and recycling of base constituents (Figure 3). Lysosomes not only represent degradative mediators of AVs but are also signaling scaffolds for AMPK and mTOR autophagy-related activities. For example, the kinase activities of mTOR are regulated by the GTPase RAS homolog enriched in brain (RHEB), which is situated on the lysosome surface, and mTOR itself has been found localized on the lysosomal compartment [92,93]. Similarly, recent investigations demonstrated that AMPK is a resident protein of lysosomes and that the lysosomal protein complex composed of vacuolar-type ATPase (V-ATPase) and Ragulator (required for the mTOR signaling pathway) is essential to phosphorylate and activate AMPK in response to nutrient starvation [94,95]. Autophagy was first described more than 50 years ago. Nevertheless, only in the last two decades have the functions of this catabolic process been elucidated, and currently, autophagy is considered one of the main mechanisms regulating the pathophysiology of many human diseases [96,97,98]. In particular, defects and alteration in the autophagic process have been associated with tumor growth, tumor suppression, cancer-drug resistance, and metastasis [99,100]. Autophagy may preserve the genomic stability, remove damaged organelles and their defective proteins after cell injury, thus battling and counteracting cancer development. Defective levels of the autophagy gene BECN1 were indeed found in human hepatocellular carcinoma and prostate, breast, and ovarian cancers [101]. Similarly, mutations in different autophagy related genes (ATG5, ATG2B, ATG16L1, and ATG9B) were observed in hepatocellular carcinoma and gastric and colorectal cancers [102]. Autophagy also prevents tumor formation by counteracting the chronic inflammatory condition typical of the tumor environment. For example, oncogenic transformation in lung cell carcinoma was correlated with increased activation of IL-6 and reduced autophagy [103]. Deficiency of ATG16L1 can provoke activation of IL-1β and IL-18 and is associated with an elevated risk of colorectal cancers [104,105].

On the other hand, autophagy is a defense mechanism that sustains the tumor metabolism and promotes tumor development and metastasis. Consistent with this idea, different studies describe increased autophagy activities in different cancer types [106,107,108,109] and correlated augmented autophagic marker levels with more aggressive tumor phenotypes [110]. Several reports show that genetic inhibition of ATG genes, such as ATG7, prevents tumor formation and the progression of colorectal, lung, and prostate cancers and glioblastoma [111,112,113]. Autophagy is also used by cancer cells to evade several cancer treatments, including radiation therapy and chemotherapy, and typical cellular stress conditions of cancer cells (hypoxia, nutrient deprivation, and metabolic stress) induce cytoprotective and pro-survival autophagy.

Autophagic dynamics in cancer are modulated by Ca^2+^ signaling. The inhibition of VGCCs induces the activation of autophagy in human adenocarcinoma and endometrial carcinoma. This process is accompanied by both increases in the apoptosis rate and decreased proliferation and migration, suggesting that intracellular Ca^2+^ is necessary for the protection of cancer cells against autophagic death [114]. ORAI1 is another protein involved in the regulation of Ca^2+^-mediated autophagy in cancer cells. It has been shown that ORAI1 downregulation delays cytoplasmic Ca^2+^ clearance, thereby promoting the activation of several Ca^2+^-dependent kinases. This activation signal impacts the expression of the cyclin-dependent kinase inhibitor p21, which results in activation of autophagy, cell growth arrest, and increased cell survival [115]. Additionally, diverse Ca^2+^-mobilizing agents (thapsigargin, ATP, ionomycin, and chemotherapic agents) [116,117] and nutrient withdrawal [118] provoke increases in [Ca^2+^]_c_ levels and the simultaneous activation of pro-survival autophagy in cancer cells. Consistently, addition of intracellular Ca^2+^ chelators prevented autophagic activation and induced cell death. Different downstream effectors were proposed to regulate Ca^2+^-dependent autophagy. For example, it was suggested that increases in [Ca^2+^]_c_ determined activation and phosphorylation of protein kinase Cθ stimulating LC3-II conversion and autophagy [119]. Rapid elevation in [Ca^2+^]_c_ was also associated to activation of the ERK pathway, which activates mitochondrial depolarization, autophagy, and apoptosis [120]. However, the primary key regulating factor of autophagy following intracellular Ca^2+^ elevation is likely CAMK2. Indeed, an increase in [Ca^2+^]_c_ promotes activation of CAMK2, which, in turn, activates autophagy through the regulation of the AMPK/mTOR pathway [116]. Importantly, it has also been demonstrated that [Ca^2+^]_c_ dynamics are regulated at ER levels by BCL-2 protein, which lower [Ca^2+^]_ER_ levels, thus reducing the Ca^2+^ leak from ER [58].

In contrast, inhibition of Ca^2+^ mobilization from the ER can also increase autophagic flux. In this scenario, IP3Rs are the primary regulator. Lithium and L-690330 stimulate autophagy by reducing the levels of IP3 and inositol and consequent IP3Rs activities [121]. Consistent with this process, the downregulation of IP3R3 levels reduces Ca^2+^ dynamics and promotes autophagy [122]. Interestingly, all these dynamics have been attributed to Ca^2+^-dependent mitochondrial activation. Correct IP3R-mediated Ca^2+^ transfer between ER and mitochondria supports the tricarboxylic acid cycle and consequent ATP production. This signal determines the inhibition of the energy sensor of the ATP/AMP ratio, AMPK, which is also the most investigated positive regulator of autophagic induction mechanism: AMPK-ULK1-mTOR [122]. Recently, it was demonstrated that the acquisition of tumor-promoting behaviors in tumors lacking the tumor suppressor protein PML relies on the IP3R3-dependent regulation of autophagy. Indeed, PML loss decreases the transfer of Ca^2+^ from the ER to mitochondria with a subsequent decrease in ATP production, which determines the activation of the AMPK pathway, thereby promoting pro-survival autophagy [100]. Similar effects were also found in renal cell carcinoma, where upregulated expression of miR-501 increases autophagy by activating AMPK. In this study, the authors demonstrated that the miR-501 decreases the activity of the MCU channel, provoking a reduction in mitochondrial activities and resulting in reduced ATP production and activation of the AMPK/ULK1 pathway [123] (Figure 3).

## 5. New Strategies for Ca^2+^ Signaling in Cancer Therapy: Ca^2+^ Channels and Pumps as Targets

Ca^2+^ is an important second messenger that regulates different cellular processes linked to cancer such as cell proliferation, apoptosis, and autophagy. Dysregulation of Ca^2+^ signaling may contribute to cancer development and expansion; therefore, targeting Ca^2+^ signaling pathways may be a good option for cancer treatment. Alterations in Ca^2+^ homeostasis may occur by impaired Ca^2+^ channel/pump expression, mutation or protein mislocalization that lead to the remodeling of various signaling pathways contributing to carcinogenesis [124]. The involvement and pharmacological targeting of Ca^2+^-related proteins associated with cancer are discussed below.

### 5.1. TRP Channels

TRPCs are classified into different subfamilies (TRPA, TRPC, TRPM, TRPML, TRPP, TRPN, and TRPV) and are implicated in different diseases, including cancer [125]. Alterations of the TRPCs (canonical) subgroup, in particular TRPC1, TRPC3, and TRPC6, are associated with a variety of cancer types, including breast, pancreatic, glioblastoma, lung, hepatic, myeloma, and thyroid cancers [126]. The dysfunction of the TRPM (melastatin) subfamily is also involved in different cancers. In particular, TRPM1 was found to be decreased in melanoma; TRPM2 is overexpressed in prostate, breast, and pancreatic cancer; and TRPM4 and TRPM5 are upregulated in prostate and lung cancers, respectively. TRPM7 is increased in breast and pancreatic cancers, while TRPM8 is markedly increased in prostate cancers and in pancreatic carcinoma [127]. Furthermore, the dysregulation of TRPV (vanilloid) channels is mostly associated with prostate cancers but is involved also in other tumors. TRPV1 and TRPV2 expression affects bladder and prostate cancer. TRPV4 is downregulated in prostate, skin, and breast cancers, while TRPV6 is boosted in various tumors, including prostate and breast cancers [125,128]. Most of these TRP channels can be targeted for cancer therapy. In this regard, the treatment of different colon cancer cell lines with 20-GPPD, a metabolite of ginseng, induces apoptosis by intracellular Ca^2+^ elevation through the activation of TRPCs. Moreover, the inhibition of TRPCs by the SKF96365 compound caused cell cycle arrest in glioblastoma cells [125,129]. The inhibition of TRPM7 channels by carvacrol treatment reduced the viability, migration, and invasion of U87 glioma cells by inactivating the RAS/MEK/MAPK and PI3K/AKT signaling pathways [130]. In addition, treatment with D-3263, an activator of TRPM8, induces the apoptosis in different cancer cell lines, decreases mice prostate hyperplasia and has been used in phase I clinical trial (https://clinicaltrials.gov/ct2/show/NCT00839631) for the treatment of various solid tumors [125]. This clinical trial not only evaluated the safety and pharmacokinetic profile of D-3263 hydrochloride in a group of patients with advanced solid tumors refractory to conventional therapy but also assessed its antitumor activity. Despite preliminary results showing disease stabilization in persons with prostate cancer [131], no recent clinical results have been reported. The activation of TRPV1 by capsaicin and TRPV2 by cannabidiol generates a continuous influx of intracellular Ca^2+^, inducing the apoptosis in prostate and bladder cancer cells, respectively [129,132]. Finally, the pharmacological targeting of TRPV6 by the peptide SOR-C13 led to the inhibition of cell growth in cellular and animal models for ovarian and prostate cancers [128]. Interestingly, SOR-C13 has been successful in a phase I study. SOR-C13 was found to be safe, well tolerated, and displayed anticancer activity in 12 of the 22 evaluable patients affected by advanced solid epithelial tumors [133].

### 5.2. VGCCs and Purinergic P2 Receptors

VGGCs are classified in high voltage activated (HVA) and low voltage activated (LVA) channels according to their pharmacological and electrophysiological profiles. They regulate various Ca^2+^-dependent cellular processes, including cell proliferation, survival, and differentiation [134]. LVA are also known as T-type Ca^2+^ channels and are frequently altered in different cancer types. The upregulation of T-type Ca^2+^ channels has been mainly observed in prostate, breast, and ovarian cancers; however, it has also been found in melanoma, retinoblastoma, glioma, glioblastoma, hepatocellular, colon, and esophageal cancers cells [134]. The pharmacological inhibition of T-type Ca^2+^ channels by using the channel blocker mibefradil reduced esophageal and colon cancer cell proliferation by upregulating p53. Moreover, this inhibitor induced the apoptosis in glioblastoma cells and ovarian cancer cells. Furthermore, the administration of mibefradil or NNC-55-096, another T-type Ca^2+^ channel blocker, decreased tumor growth in xenograft models of glioblastoma and ovarian cancers [134].

Purinergic P2 receptors are classified in two subfamilies named P2X and P2Y. P2X receptors, activated by ATP, are ligand-gated nonselective cation channels formed by homotrimeric or heterotrimeric complexes of seven different subunits (P2X1–7). The P2Y receptor category, comprising eight members (P2Y1, P2Y2, P2Y4, P2Y6, and P2Y11–14), may be activated by ATP, ADP, UTP, UDP, and UDP-glucose. In particular, P2Y2, P2Y4, and P2Y6 receptors are coupled to Gq proteins and their stimulation leads to Ca^2+^ mobilization by the activation of IP3Rs and SOCE channels [10]. In cancer cells, P2X7R dysfunction impairs the ability of this receptor to open the macropore in response to high extracellular ATP concentration present in the tumor microenvironment, preventing prolonged plasma membrane depolarization and cell death. Moreover, many tumor types including prostate, lung, kidney, colorectal, gastric, and breast cancers, express mutated forms of P2X7R, which are associated with tumor development, survival, and metastasis [135]. Furthermore, P2X3R and P2X5R were found to be overexpressed in hepatocellular carcinoma and squamous cell carcinoma, respectively [10]. Among P2Y family members, P2Y2 is upregulated in breast, hepatoma, pancreatic adenocarcinoma, and colon cancers while P2Y4 is overexpressed in colon cancers [125]. Many anti-P2X7R molecules have been developed in order to treat different diseases, including cancer. Among them, BIL010t and BIL06v, which have been tested in basal cell carcinoma (BCC) and other solid tumors, seem to be the most promising therapeutics [135]. Consistently, a phase I clinical trial demonstrates that BIL010t is safe, tolerable, and reduces primary lesions of BCC [136], meanwhile the phase I study for BIL06v in advanced tumors is ongoing (registration number: ACTRN12618000838213). The pharmacologic inhibition of P2RY2 by using its selective antagonist AR-C118925XX reduces tumor cell growth in xenograft models of pancreatic ductal adenocarcinoma [137].

### 5.3. SOCE Machinery Proteins (ORAI and STIM)

ORAI channels and STIM Ca^2+^-sensors are molecular SOCE components. Currently, in mammalian cells, three isoforms of ORAI (ORAI1, ORAI2, and ORAI3) and two isoforms of STIM (STIM1 and STIM2) have been identified. Remodeling of Ca^2+^ signals due to SOCE dysregulation may cause various diseases, including cancer. In fact, activating ORAI1 mutations were found in different types of cancer, including colorectal, stomach, and uterine cancers [138]. Moreover, increased expression of ORAI1 and STIM1 is involved in glioblastoma, pancreatic adenocarcinoma, and breast, prostate, liver, and kidney cancers [125]. Altered expression of STIM2 was found in melanoma and colorectal cancers, while high levels of ORAI2 were observed in acute myeloid leukemia cell lines [138]. Nevertheless, the channel most involved in carcinogenesis is ORAI3, which is expressed in mammalian cells only. Increased levels of ORAI3 form an SOC channel that drives tumorigenesis in estrogen receptor-positive breast cancer as well as in lung adenocarcinoma. Moreover, the interaction between ORAI3 and ORAI1 leads to the generation of arachidonic/leukotriene-regulated heteromeric Ca^2+^ channels expressed in prostate and colorectal cancers but not in healthy tissue [139,140].

The first described inhibitor for ORAI1 channel was SKF-96365, which is able to reduce the growth and migration of breast cancer cells [139]. SOCE channels are also inhibited by trivalent ions such as La^3+^ and Gd^3+^; however, these channel blockers, as well as SKF-96365, are not SOCE-specific inhibitors; therefore, treatment with these compounds may cause side effects. In this regard, DPB-162AE and DPB-163AE, derivatives of 2-APB, have been developed and are potent SOCE inhibitors capable of inhibiting SOC channels without affecting IP3Rs activity. In addition, RO2959, which inhibits the ORAI1-mediated current, may represent an important therapeutic tool since it is able to selectively increase the ORAI1 channel [141]. Another promising molecule for cancer treatment is ML-9, which inhibits SOCE, blocking STIM1 plasma membrane translocation. This compound administered alone or in combination with other drugs induces prostate cancer cell death [125]. As these compounds have been tested only in cellular models, future studies will be needed to corroborate their effectiveness in cancer therapy. Some drugs with anticancer properties including rapamycin and its analogs, are able to inhibit STIM1- and ORAI1-dependent Ca^2+^ influx. These mTOR inhibitors are being tested in different clinical trials as anticancer therapy [141]. ORAI1-dependent Ca^2+^ influx was also found to be crucial for activating the cell death induced by the anti-CD20 monoclonal antibody GA101/obinutuzumab in non-Hodgkin lymphoma and primary B-cell chronic lymphocytic leukemia cells. Moreover, in addition to ORAI1-dependent Ca^2+^ influx, in this study, it was demonstrated that GA101 determines intracellular Ca^2+^ elevation by provoking Ca^2+^ release from lysosomes [142].

### 5.4. IP3Rs and Ca^2+^-ATPases

IP3Rs consist of three isoforms (IP3R1, IP3R2, and IP3R3) that are activated by the generation of intracellular IP3. The most isoform of IP3 receptor isoforms involved in carcinogenesis is IP3R3. The dysfunction of this receptor was found in clear cell renal cell carcinoma cells and in colorectal and ovarian cancer cell lines, where this receptor exerted proliferative and antiapoptotic effects [143]. Furthermore, the upregulation of IP3R2 seems to be associated with the growth of chronic lymphocytic leukemia cells [144]. Only a few IP3Rs inhibitors have been tested in cancer models. However, the caffeine treatment of “in vitro” and “in vivo” models of glioblastoma inhibited cell migration, and it increased the survival of a mouse xenograft model of glioblastoma by inhibiting the IP3R3 receptor channel [145].

Altered expression or mutations of SERCA isoforms (SERCA2 and SERCA3) was observed in several cancer types, including colon, gastric, lung, and prostate carcinoma [125,146,147]. The upregulation of SPCA1 and the translocation of SPCA2 to the plasma membrane were found in breast cancers, where these pump types seem to promote Ca^2+^-dependent cell proliferation [148]. In addition, PMCA isoforms are dysregulated in various cancer types. In particular, PMCA2 is overexpressed in different breast cancer cell lines, while PMCA1 is upregulated in colon cancer cells but downregulated in oral squamous cell carcinoma cell lines. Moreover, the expression of PMCA4 was found to be reduced in colon cancer cells and in breast and colon cancers tissues [149,150]. In contrast, this pump was found to be overexpressed in different pancreatic ductal adenocarcinoma tumors, where it correlates with poor patient survival [151,152]. However, the different expression levels of PMCA ATPases observed in various cancers suggests that these pumps can function in different ways depending on the tumor type. Nevertheless, the contribution of these molecules to cancer development and progression remains unclear and needs further investigation.

In the last years, many compounds able to inhibit SERCA for cancer treatment have been produced. Mipsagargin (G-202), a thapsigargin derivate, has been tested on different solid tumors, including prostate cancers, glioblastoma, kidney, and hepatocellular carcinoma, in phase I and II clinical trials. The results obtained in phase I demonstrate that mipsagargin displays a favorable pharmacokinetic profile and acceptable tolerability. Furthermore, significant disease stabilization was observed, suggesting possible antitumor activity [153]. The results obtained in phase II supported the hypothesis of antitumor activity and demonstrated that mipsagargin induces prolonged disease stabilization in patients affected by hepatocellular carcinoma and may represent an effective therapeutic treatment for advanced tumors [154]. Treatment with curcumin, another SERCA inhibitor, promotes apoptosis of cells derived from various tumors, such as breast, lung, ovarian, and colon cancers [155]. In addition, PMCA inhibitors were developed for anticancer therapy. In fact, treatment with the selective PMCA inhibitor [Pt(O,O0-acac)(γ-acac)(DMS)] induced the apoptosis of MCF7 breast cancer cells by elevating cytosolic Ca^2+^ levels [156]. Furthermore, resveratrol and its derivatives reduce cell viability through the increase of intracellular Ca^2+^ levels by inhibiting PMCA in prostate cancer cells. Unfortunately, the function of the latter compounds is exerted by the activation of IP3Rs; therefore, they cannot be considered PMCA-specific inhibitors [125,157].

### 5.5. MCU and VDAC

Alterations in the MCU complex expression/function were found in different cancer types. High expression levels of MCU were detected in colorectal, ovarian, pancreatic, stomach, and prostate cancers, while genetic mutations were observed mainly in prostate, breast, and uterine cancers. Genetic modifications linked to cancer development were also detected in the other components of the MCU complex [158]. Based on these observations, the targeting of MCU channel for cancer therapy may be an intriguing option, especially for cancer patients who overexpress the MCU protein. However, compounds able to inhibit this channel, including ruthenium red and its derivative ruthenium 360, are nonspecific and lead to different side effects. Recently, a new membrane-permeant MCU complex inhibitor named DS16570511 was identified, but its anticancer properties need further investigation [158].

Another important mitochondrial protein is VDAC; this pore is a nonselective Ca^2+^-permeable pore located on the outer membrane of mitochondrion, where it regulates the flux of ions and metabolites from cytosol to mitochondria and vice versa. Three isoforms for this channel, VDAC1, VDAC2, and VDAC3, have been identified in mammalian cells [159]. Based on the assumption that VDAC pores regulate mitochondrial Ca^2+^ fluxes, it is speculated that VDACs may be involved in the control of cell proliferation and apoptosis; therefore, these channels may affect the fate of cancer cells. VDAC1 is upregulated in a variety of human cancer cell lines, while VDAC2 is overexpressed in melanoma, mesothelioma, and thyroid cancer cells [159]. The targeting of VDAC isoforms may be an important option for cancer treatment. In fact, the administration of R-Tf-D-LP4, a VDAC-based peptide, in xenograft mouse models of glioblastoma, lung, and breast cancer inhibited tumor growth, causing massive cancer cell death [160].

## 6. Plan of Action in Cancer Therapy: Intersection between Cancer Therapies with Ca^2+^ Signaling

Proliferation, invasiveness, cell death, neovascularization, gene transcription, protein production, and phosphorylation/dephosphorylation events are some of the numerous targets of anticancer compounds. Given that Ca^2+^ signaling is extensively involved in these molecular processes, it is not surprising that an anticancer agent may indirectly modulate Ca^2+^ dynamics in cancer cells. Various studies demonstrated that chemotherapeutic agents modulate intracellular Ca^2+^ levels. For example, 5-fluorouracil (5FU) is an approved anticancer treatment for several cancer types. It has been observed that 5FU mediates in hepatocarcinoma cell death by diminishing Ca^2+^ influx. Indeed, 5FU administration decreased ORAI1 levels and induced autophagic cell death by inhibiting PI3K/AKT/mTOR pathway [161]. In contrast, in colon carcinoma cells, 5FU mediated its cytotoxic effects by increasing intracellular Ca^2+^ amounts to a level necessary to activate calmodulin, which, in turn, phosphorylated p53 to trigger apoptosis [162]. Similar effects were also found with the clinical chemotherapeutic agent cisplatin, which initiated ER stress, the unfolded protein response and Ca^2+^-mediated apoptosis [163]. Additionally, dexamethasone and other glucocorticoid hormones used for the treatment of lymphoid malignancies increased intracellular Ca^2+^ transport. However, in this case, Ca^2+^ dynamics were associated with chemoresistance. Indeed, both inhibition of TRPCs and Ca^2+^ chelation increased the sensitivity of human leukemia cells to dexamethasone [164,165]. Studies on the chemotherapeutic drugs doxorubicin and simvastin (belonging to the anthracyclines family) reported a direct effect on Ca^2+^ signaling. Accordingly, these drugs induced the persistent release of Ca^2+^ from intracellular stores, provoking mitochondrial Ca^2+^ accumulation and apoptosis. Interestingly, doxorubicin also promoted the binding of p53 to SERCA in the ER. In this state, p53 increased Ca^2+^ transmission between the ER and mitochondria to induce apoptosis [166]. Taxane paclitaxel is widely used in clinical practice for ovarian, breast, neck and head cancers. Paclitaxel induces cytosolic Ca^2+^ oscillations that affect neuronal Ca^2+^ sensor 1 proteins, leading to Ca^2+^ release from the ER via an IP3R3-dependent pathway [167]. Photodynamic therapy (PTD) refers to the use of photosensitizing agents to kill cancerous cells by generating oxidative stress capable of causing damage to cell membranes, proteins, and/or DNA. PTD may promote its anticancer effects by increasing intracellular Ca^2+^ concentration and activating the apoptotic pathway in a p53-dependent pathway [168]. Finally, there is mounting preclinical evidence showing that modulating the autophagic response may improve the efficacy of conventional anticancer drugs for late-stage tumors. Intriguingly, a growing body of evidence highlights that a series of autophagic inhibitors modulate Ca^2+^ signaling in tumors, particularly 4-aminoquinoline antimalarial compounds chloroquine (CQ) and hydroxychloroquine (HCQ). Currently, more than 30 clinical studies are evaluating the antitumor efficacy of CQ and HCQ. Publications reporting the clinical trial results are encouraging. Indeed, most of these investigations describe positive and/or partial effects of CQ and HCQ in reducing tumor growth alone or in combination with the conventional therapies used for several cancer types [169]. It has been demonstrated that CQ decreased the intracellular Ca^2+^ accumulation by inhibiting the IP3Rs-dependent ER Ca^2+^ release and the Ca^2+^ influx mediated by TRPCs, ORAI, and STIM channels [170]. This effect was found in primary B lymphocytes, suggesting that CQ cooperate with Ca^2+^ signaling to modulate the immunological response [171]. This hypothesis was confirmed in a recent study showing that CQ drives the switch of tumor-associated macrophagy (TAM) from the M2 phenotype to the tumor-killing M1 phenotype. In this scenario, CQ increased the intracellular Ca^2+^ levels that were necessary to activate p38, NF-kB, and TFEB to reprogram the TAM phenotype [171]. CQ exerts its anticancer effects by modulating Ca^2+^ homeostasis also in solid tumors associated with PML absence or downregulation. Loss of PML conferred resistance to chemotherapies due to a reduction in ER-mitochondria Ca^2+^ transmission that activates autophagy and establishes a metabolic advantage for the cancer cells. As a consequence of blocking autophagy with the specific inhibitors (CQ, 3-methyladenine or siRNA BECN1), the apoptotic process was rescued in vitro and in vivo [100]. Similar results were obtained in glioblastoma cells, where CQ promoted impairment in protein folding, ER stress, subsequent Ca^2+^ release, and activation of apoptosis. Interestingly, specific MCU inhibitors or MCU silencing abrogated CQ-dependent effects, thus confirming the importance of mitochondrial Ca^2+^ overload for cell death induction [172]. A reduction in tumor growth and the activation of cell death was observed upon the inhibition of essential autophagy-related genes. Consistent with this finding, knocking down ATG5 led to recovered Ca^2+^ mobilization in glioma cells that had previously been rendered sensitive to anticancer therapy [173]; in addition, the genetic ablation of ATG7 in renal cell carcinoma counteracts the excessive autophagic level caused by a reduction in mitochondrial Ca^2+^ uptake and ATP production and diminishing cancer cell proliferation and migration [123]. Another emerging approach to counteract tumor-promoting conditions is cancer immunotherapy, which improves the cancer-killing efficiency of tumor-infiltrating T lymphocytes (TILs). A characteristic of TILs is the expression of the cell surface receptor programmed death-1 (PD-1) [174]. PD-1 binding to its ligands, PD-L1 or PD-L2, inhibits the activation of T cells. Antibodies blocking the PD-1/PD-L1 signaling pathway reactivate the T-cell-mediated immune response and are employed for the treatment of patients with cancer. Unfortunately, some patients initially respond to immunotherapy but then suffer rapid disease progression. These antibodies, such as pembrolizumab, also modulate intracellular Ca^2+^ signaling, which improves the chemotaxis of T cells by increasing intracellular Ca^2+^ influx [175]. Furthermore, it has been shown that Ca^2+^ signaling inhibits PDL1 and PDL2 expression [176] and that Ca^2+^ flux is abolished when T cells express high levels of PD-1 [177]. Therefore, cancer immunotherapy may be further improved by coupling commonly used antibodies blocking PD-1/PD-L1 signaling and regulators of Ca^2+^ transmission. However, a greater understanding of the steps involving Ca^2+^ signaling during cancer immunotherapy is required.

## 7. Discussion

Cellular Ca^2+^ is a ubiquitous signal that contributes to the control of diverse cellular functions. Uncontrolled remodeling of Ca^2+^ flux contributes to severe pathophysiology processes and often intersects key aspects of cancer progression, such as tumor proliferation, malignant transformation, escape from cell death, and resistance to anticancer agents. Accumulating preclinical and clinical evidence supports the relationship between Ca^2+^ and cancer, indicating that Ca^2+^ signaling is a reliable target for novel anticancer treatments. As summarized in this review, defects in Ca^2+^ channels/transporters/pumps are typical features of cancerous cells and confer low sensitivity to cell death inducers, thus sustaining the tumor growth and metastasis. Hence, pharmacological modulation of these proteins may be a reliable approach to restore the effectiveness of current cancer treatment regimes (Table 1). However, before thinking about an effective therapeutic intervention based on pharmacological modulation of Ca^2+^-regulators, it is important to consider other critical aspects. Targeting these processes is difficult, and most importantly, the tumor environment presents substantial cellular heterogeneity in which only a subset of cancer cells should be targeted. Recent investigations have made progress in overcoming these problems. For example, by encapsulating pumps and channel agonists in lipid nanocapsules, it is possible to efficiently modulate the activities of these pumps and channels and the related cellular processes [178]. In addition, by coupling these agents to peptides to create a prodrug that is activated only by a cancer-specific protease, the cytotoxic effects of Ca^2+^ modulators can be solely directed to the cancer cell population [179,180]. Further studies are needed to verify the effective toxicity and pharmacokinetic of these modulators prior to performing clinical testing. Autophagy has also attracted attention in the cancer context. It supplies nutrients to the tumor, suppresses the immune response, and helps cancer cells evade cell death and conventional chemotherapy. Despite the interconnections between autophagy and Ca^2+^ in cancer, this area of study is still in its infancy, with a number of studies starting to explore and highlight the importance of these interconnections. Removing the remaining gap in our knowledge on the intersections between Ca^2+^ and cancer will help researchers better understand the multiple molecular mechanisms that affect tumor development, maintenance, and metastasis and help clinicians design and develop new-generation drugs with the final aim of breaking all the defense barriers of cancer.

## Figures and Tables

**Table 1:** Summary of the main compounds targeting Ca^2+^ channels/transporters/pumps.

Channel/Transporter/Pump	Compound	Cancer
**TRPCs**	20-GPPD	Colorectal
	SKF96365	Glioblastoma
	Carvacrol	Glioma
	D-3263	Prostate, colon, breast, lung, pancreas, leiomyosarcoma, and Kaposi’s sarcoma
	Capsaicin	Prostate
	Cannabidiol	Bladder
	SOR-C13	Ovarian and prostate
	Dexamethasone	Leukemia
**VGGCs**	Mibefradil	Esophageal, colon, glioblastoma, and ovarian
	NNC-55-096	Glioblastoma and ovarian
**Purinergic P2 receptors**	BIL010t	Basal cell carcinoma
	BIL06v	Advanced or metastatic solid tumors
	AR-C118925XX	Pancreatic ductal adenocarcinoma
**ORAI and STIM**	SKF96365	Breast
	DPB-162AE/-163AE	Colon and glioma
	ML-9	Prostate
	GA101/obinutuzumab	Non-Hodgkin lymphoma and leukemia
	5-Fluorouracil	Hepatocarcinoma
**SERCA**	Mipsargargin	Prostate cancers, glioblastoma, kidney, and hepatocellular carcinoma
	Curcumin	Breast, lung, ovarian, and colon
**PMCA**	Pt(O,O0-acac)(γ-acac)(DMS)	Breast
	Resveratrol	Prostate
**IP3R3**	Paclitaxel	Ovarian, breast, neck and head
**VDAC**	R-Tf-D-LP4	Glioblastoma, lung, and breast

## Abbreviations

AMPK	5′ adenosine monophosphate-activated protein kinase
APs	autophagosomes
ATG	autophagy-related
BAP1	BRCA1-associated protein 1
BCL-2	B-cell lymphoma 2
BECN1	coiled-coil, moesin-like BCL2 interacting protein
Ca^2+^	calcium
Cyt-C	cytochrome C
CAM	calmodulin
CAMK	Ca^2+^/Calmodulin Dependent Protein Kinases
CaN	calcineurin
CDK	cyclin-dependent protein kinases
DISC	death-inducing signaling complex
ER	endoplasmic reticulum
HVA	high voltage activated
IP3Rs	inositol 1,4,5-triphosphate receptors
LC3	microtubule-associated proteins 1A/1B light chain 3
LVA	low voltage activated
MAMs	mitochondria associated membranes
MAPK	mitogen-activated protein kinase
MCU	mitochondrial calcium uniporter
MPT	mitochondrial permeability transition
mTOR	mammalian target of rapamycin
NCX	Na^+^/Ca^2+^ exchanger
NFAT	nuclear factor of activated T-cell
NMDA	N-methyl-d-aspartate receptor
PCD	programmed cell death
PD-1	programmed death-1
PI3K	phosphatidylinositol 3-kinase
PI3P	phosphatidylinositol-3-phosphate
PM	plasma membrane
PMCA	plasma membrane Ca^2+^ transport ATPase
PML	promyelocytic leukemia protein
PTEN	protein phosphatase and tensin homolog
RHEB	RAS homolog enriched in brain
RIPK	receptor-interacting serine/threonine-protein kinase
RyRs	ryanodine receptors
SERCA	sarcoendoplasmic reticulum Ca^2+^-ATPase
SOCE	store-operated Ca^2+^ entry
SPCA	secretory protein calcium ATPase
TFEB	transcription factor EB
TIL	tumor-infiltrating T lymphocytes
TRPML1	transient receptor potential mucolipin 1
TRPC	transient receptor channels
ULK	Unc-51 like autophagy activating kinase
V-ATPase	vacuolar-type ATPase
VDAC	Voltage-dependent anion channels
VGCC	voltage-gated Ca^2+^ channels
